# Development of an Influenza Rapid Diagnostic Kit Specific for the H7 Subtype

**DOI:** 10.3389/fmicb.2018.01346

**Published:** 2018-06-25

**Authors:** Kiyoko Iwatsuki-Horimoto, Jianzhong Shi, Xiurong Wang, Yuko Sakai-Tagawa, Mutsumi Ito, Kazushi Murakami, Tiago J. da Silva Lopes, Kazunari Nakaishi, Seiya Yamayoshi, Satoshi Watabe, Hualan Chen, Yoshihiro Kawaoka

**Affiliations:** ^1^Division of Virology, Department of Microbiology and Immunology, Institute of Medical Science, University of Tokyo, Tokyo, Japan; ^2^State Key Laboratory of Veterinary Biotechnology, Harbin Veterinary Research Institute, Chinese Academy of Agricultural Sciences, Harbin, China; ^3^TAUNS Laboratories, Inc., Shizuoka, Japan; ^4^Influenza Research Institute, Department of Pathobiological Sciences, School of Veterinary Medicine, University of Wisconsin–Madison, Madison, WI, United States; ^5^Department of Special Pathogens, International Research Center for Infectious Diseases, Institute of Medical Science, University of Tokyo, Tokyo, Japan

**Keywords:** influenza virus, rapid diagnostic kit, H7 subtype, highly pathogenic avian influenza, monoclonal antibody

## Abstract

Since the spring of 2013, human infections with H7N9 viruses have been detected in China. Some of these viruses have become highly pathogenic. Highly and low pathogenic avian influenza H7N9 viruses are currently co-circulating with the seasonal influenza A viruses H3N2 and H1N1pdm09. Prompt identification and isolation of H7N9 patients is one measure to prevent the spread of H7N9 virus and help prevent a pandemic. The majority of commercially available point-of-care rapid influenza diagnostic kits can differentiate between influenza A and B viruses, but cannot distinguish between H7N9 viruses and seasonal influenza A viruses. Accordingly, we have developed a rapid diagnostic kit specific for the H7 subtype that is accessible, easy to use. Although the detection limit of this H7 kit is one-tenth lower than that of a commercially available rapid influenza A and B diagnostic kit of similar design, except for the specificity of the monoclonal antibodies used, this kit is highly specific, detecting only H7-subtype influenza viruses, including the recent highly pathogenic H7N9 viruses from humans, and does not show any non-specific reactions with other HA subtypes. This H7 kit will be of value for the early detection of H7N9-infected patients.

## Introduction

Human infections with low pathogenic avian influenza (LPAI) H7N9 virus were first reported in the spring of 2013 in China (Centers for Disease Control and Prevention, 2013; Gao R. et al., 2013). As of 13 February 2018, 1625 human cases of infection and 621 deaths have been attributed to this virus (Food and Agriculture Organization of the United Nations, 2018). The major sources of these human cases are believed to be H7N9 virus-infected live poultry or contaminated environments, especially live poultry markets (Gao R. et al., 2013; Shi et al., 2013; Zhang et al., 2013; Yu et al., 2014; Lam et al., 2015).

In the 2016–2017 influenza season, the fifth and largest wave of LPAI H7N9 occurred in southern China (World Health Organization, 2017c). In addition, highly pathogenic avian influenza (HPAI) H7N9 viruses emerged and infected humans during the fifth wave (World Health Organization, 2017b). Phylogenetically, the HPAI H7N9 viruses were derived from the LPAI H7N9 viruses circulating among domestic poultry (Ke et al., 2017; Shi et al., 2017; Zhang et al., 2017). Although sustained human-to-human transmission of the virus has not yet been reported, several mammalian-adaptive mutations have been detected in H7N9 viruses (Wang D. et al., 2014; Wang Y.R. et al., 2014; Watanabe et al., 2014; Xiao et al., 2016). These mutations may contribute to the ability of these viruses to infect mammals. Shi et al. (2017) found that the H7N9 HPAI readily obtained the 627K or 701N mutation in its PB2 segment upon replication in ferrets, causing it to become highly lethal in mice and ferrets and to be transmitted efficiently in ferrets by respiratory droplet. In addition, we found that HPAI H7N9 viruses isolated from humans are able to transmit among ferrets (Imai et al., 2017). If H7N9 viruses gain the ability to transmit efficiently from human-to-human, they could cause a pandemic. In China, seasonal influenza A viruses, H3N2 and H1N1pdm09, are co-circulating with HPAI and LPAI H7N9 viruses (China CDC, 2017). The continued circulation of these viruses increases the possibility for not only the incorporation of further human adaptive-mutations but also for reassortment with circulating human viruses of the H1N1pdm09 or H3N2 subtypes. H7N9 viruses thus pose a potential pandemic threat.

Prompt identification and isolation of H7N9 patients is one means to prevent the spread of H7N9 virus. However, we cannot distinguish influenza virus subtypes based on the symptoms of patients. Although the severity of H7N9 virus infection is generally higher than that of seasonal H3N2 and pdmH1N1 virus, but lower than that of H5N1 virus, the initial symptoms of human infection with avian H7N9 virus are similar to those caused by other subtypes (Gao H.N. et al., 2013; Yang et al., 2013; Yu et al., 2013). In addition, asymptomatic or mild infection of humans with H7N9 virus has also been reported (Cowling et al., 2013; Yu et al., 2013; Chen et al., 2014; Watanabe et al., 2014). Although rapid influenza diagnosis kits are commercially available, they cannot differentiate between H7N9 viruses and seasonal influenza viruses. To make a definite diagnosis, we currently need to perform real-time PCR, which requires specialized equipment and facilities; such equipment and facilities are not universally available at the bedside. In this study, we developed a rapid diagnostic test that is specific for the H7 subtype and is easy to use, and does not require special equipment. We also report our analysis of the performance of this kit with various H7N9 isolates.

## Materials and Methods

### Ethics and Biosafety Statements

The research protocol for experiments with mice was approved by and is in accordance with the TAUNS Laboratories, Inc., Shizuoka, Japan (approval number: 201306FLUH7). We used swabs from two healthy volunteers under a research protocol approved by the Research Ethics Review Committee of the Institute of Medical Science, University of Tokyo (approval number 26-42-0822). Written informed consents were obtained from both subjects. All experiments with H7N9 viruses were performed in biosafety level 3 (BSL3) laboratories at the University of Tokyo (approved for such use by the Ministry of Agriculture, Forestry, and Fisheries, Japan) and at the Harbin Veterinary Research Institute, the Chinese Academy of Agricultural Sciences (approved by the Review Board of Harbin Veterinary Research Institute, China).

### Preparation of H7 Antigen to Produce MAbs Against H7 HA

A/Anhui/1/2013(H7N9) (Anhui/1) virus was engineered by using reverse genetics as described previously (Neumann et al., 1999). Briefly, the cDNA of each of the eight segments of viral RNA of Anhui/1 were synthesized based on the sequences in GISAID [Global Initiative on Sharing All Influenza Data (accession numbers; PB2, EPI439504; PB1, EPI439508; PA, EPI439503; HA, EPI439507; NP, EPI439505; NA, EPI439509; M, EPI439506; NS, EPI439510)]. The resulting products were cloned into pHH21 plasmids. All constructs were subsequently sequenced to verify the absence of unwanted mutations. Then, Anhui/1 was generated by use of plasmid-based reverse genetics. Transfectant virus was collected and amplified in Madin–Darby canine kidney (MDCK) cells, cultured in Eagle’s minimal essential medium (MEM) containing 0.3% bovine serum albumin (BSA) and 0.75 μg/ml tosylsulfonyl phenylalanyl chloromethyl ketone (TPCK)-trypsin. Amplified Anhui/1 virus was purified through a 10–50% sucrose density gradient and resuspended in phosphate-buffered saline (PBS). To inactivate Anhui/1, β-propiolactone (final concentration, 0.1%; WAKO, Osaka, Japan) was added to the viruses, and incubated at 4°C for 16–24 h. Inactivation of Anhui/1 was confirmed by passaging the viruses twice in 10-day-old specific pathogen-free embryonated chicken eggs and then performing hemagglutination assays.

### Development of MAbs for the H7 Kit

To produce monoclonal antibodies (MAbs), 7-week-old female BALB/c mice (Charles River Laboratories, Yokohama, Japan) were immunized with inactivated Anhui/1 plus an adjuvant (unpublished data) four times at 2-week intervals and boosted at 5 or 6 days after the final immunization. Two or three days after boosting, hybridoma cell lines were established with the spleen cells of the immunized mice and myeloma cells (Sp2/O-Ag14) by using methods previously described (Yen et al., 1979). The reactivity of the hybridoma cell lines with purified HA proteins of A/California/07/2009(H1N1pdm09), A/Canada/720/2005(H2N2), A/Perth/16/2009(H3N2), A/Indonesia/5/2005(H5N1), A/northern shoveler/California/HKWF115/2007(H6N1), A/Anhui/1/2013(H7N9), and A/Hong Kong/358 20/2009(H9N2) viruses (details of the purified HA proteins are shown in Supplementary Table 1) was assessed by use of an ELISA.

### Viruses

Influenza viruses of various subtypes (**Table 1**): A/Chicken/Egypt/119S-NLQP/2011(H5N1) was kindly provided by Dr. A.-S. Arafa, Animal Health Research Institute, Egypt; A/duck/Gunma/466/2011(H7N9) was kindly provided by Dr. T. Saito, National Institute of Animal Health, Japan; A/Anhui/1/2013(H7N9) and A/Shanghai/1/2013(H7N9) were kindly provided by Dr. M. Tashiro, National Institute of Infectious Diseases, Japan; A/Guangdong/17SF003/2016(H7N9) and A/Taiwan/1/2017(H7N9) were kindly provided by Dr. T. Odagiri, National Institute of Infectious Diseases, Japan; A/duck/Hong Kong/301/78(H7N2) and A/seal/Massachusetts/1/80(H7N7) were kindly provided by Dr. H. Kida, Hokkaido University, Japan; all other viruses were isolated in our laboratory) were propagated in MDCK cells with MEM containing 0.3% BSA and 0.75 μg/ml TPCK-trypsin. The titers of these viruses were determined by using 50% tissue culture infectious dose (TCID_50_) in MDCK cells (World Health Organization, 2002). Recent H7N9 viruses isolated from birds and the environment in China (**Table 2**) were propagated in the allantoic cavity of 10-day-old embryonated chicken eggs. The titers of these viruses were determined by using 50% egg infectious dose (EID_50_) in embryonated chicken eggs. The EID_50_ was calculated by using the method of Reed and Muench (1938).

**Table 1:** Detection specificity and sensitivity of the H7 kit for various subtypes^a^.

Virus strain	Type or subtype	Detection limit (log_10_ TCID_50_/100 μl)	
		ImunoAce Flu^b^	H7 kit (highest titer tested)
A/Yokohama/UTK2A/2011	H1N1pdm	3.0	–^c^ (6.5)
A/Osaka/UT-A01/2013	H1N1pdm	2.0	– (6.3)
A/Yokohama/UT-K4A/2011	H3N2	3.7	– (6.7)
A/Tokyo/UT-IMS6-1/2013	H3N2	3.7	– (6.3)
A/Vietnam/UT36285I/2010	H5N1	3.7	– (7.3)
A/Chicken/Egypt/119S-NLQP/2011	H5N1	4.0	– (7.7)
B/Yokohama/UT-K1A/2011	B (Victoria)	3.7	– (6.5)
B/Yokohama/UT-K31/2012	B (Yamagata)	4.7	– (7.7)
A/duck/Gunma/466/2011	H7N9	4.7	6.7
A/Anhui/1/2013	H7N9 (LPAI)	4.0	5.7
A/Shanghai/1/2013	H7N9 (LPAI)	3.7	7.3
A/Guangdong/17SF003/2016	H7N9 (HPAI)	5.0	7.0
A/Taiwan/1/2017	H7N9 (HPAI)	4.5	7.0
A/duck/Hong Kong/301/78	H7N2	3.0	5.7
A/seal/Massachusetts/1/80	H7N7	3.7	5.7


*^a^Tenfold serially diluted viruses (10^1^–10^6^ TCID_50_/100 μl) or undiluted virus solution were tested. One hundred microliters of sample was mixed with 700 μl of the extraction buffer (TAUNS Laboratories, Inc.). Three drops (80–120 μl) of the mixed solution were dropped on to the sample pad of the kit. The minimum viral titers required for a positive reaction were determined twice independently. Average titers are shown. ^b^The specificity and sensitivity of the H7 kit was compared with that of the commercially available influenza rapid diagnostic kit ImunoAce Flu (TAUNS Laboratories, Inc.), which can detect both influenza A and B viruses. ^c^–, not detected.*

**Table 2:** Detection sensitivity of the H7 kit for recent H7N9 viruses isolated from birds in China^a^.

Virus strain	Detection limit (log_10_ EID_50_/100 μl)	
	ImunoAce Flu^b^	H7 kit
A/pigeon/Shanghai/S1069/2013	5.0	6.0
A/pigeon/Jiangsu/SD001/2013	6.0	7.0
A/chicken/Zhejiang/S4008/2013	5.0	6.0
A/chicken/Ningxia/S1152/2014	7.0	8.0
A/chicken/Xinjiang/SD033/2014	5.5	7.0
A/chicken/Guangdong/S4021/2014	6.0	7.0
A/duck/Fujian/S4170/2014	5.0	7.0
A/duck/Zhejiang/S4488/2014	5.0	6.0
A/environment/Hunan/SD009/2015	5.0	7.0
A/duck/Shanghai/SD016/2015	5.0	6.0


*^a^Tenfold serially diluted viruses (10^1^–10^6^ EID_50_/100 μl) or undiluted virus solution were tested. One hundred microliters of sample was mixed with 700 μl of the extraction buffer (TAUNS Laboratories, Inc.). Three drops (80–120 μl) of the mixed solution were dropped on to the sample pad of the kit. The minimum viral titers required for a positive reaction were determined three times independently. Average titers are shown. ^b^The sensitivity of the H7 kit was compared with that of the commercially available influenza rapid diagnostic kit ImunoAce Flu (TAUNS Laboratories, Inc.), which can detect both influenza A and B viruses.*

### Swabs

We tested six frozen swabs (four from chickens and two from the surrounding environment) that were collected between 2014 and 2015 in China as part of our surveillance activities. H7N9 viruses were isolated from all six frozen swabs (data not shown). Six other swabs, collected in 2013 in Indonesia, were used to demonstrate the lack of reactivity with the sample matrix: two from chickens, two from ducks, and two from quails. No viruses were isolated from these six swabs (data not shown). We also tested clinical swabs from two healthy volunteers as negative controls.

### Testing of the H7 Kit

The sensitivity and specificity of the H7 kit was compared with that of the commercially available influenza rapid diagnostic kit ImunoAce Flu (TAUNS Laboratories, Inc., Shizuoka, Japan), which can detect both influenza A and B viruses. Tenfold serially diluted (with MEM containing 0.3% BSA or PBS) viruses (10^1^–10^6^ TCID_50_/100 μl or 10^1^–10^6^ EID_50_/100 μl) or undiluted virus solution were tested. One hundred microliters of sample was mixed with 700 μl of the extraction buffer (TAUNS Laboratories, Inc.). Three drops (80–120 μl) of the mixed solution were then dropped on to the sample placement region of the kit. Results were visualized after 3–8 min of incubation at room temperature. The minimum viral titers required for a positive reaction were determined twice or three times independently. The average titers of these two or three experiments were then considered the detection limit of the kit.

## Results

### Development of the H7 Kit

**FIGURE 1:**
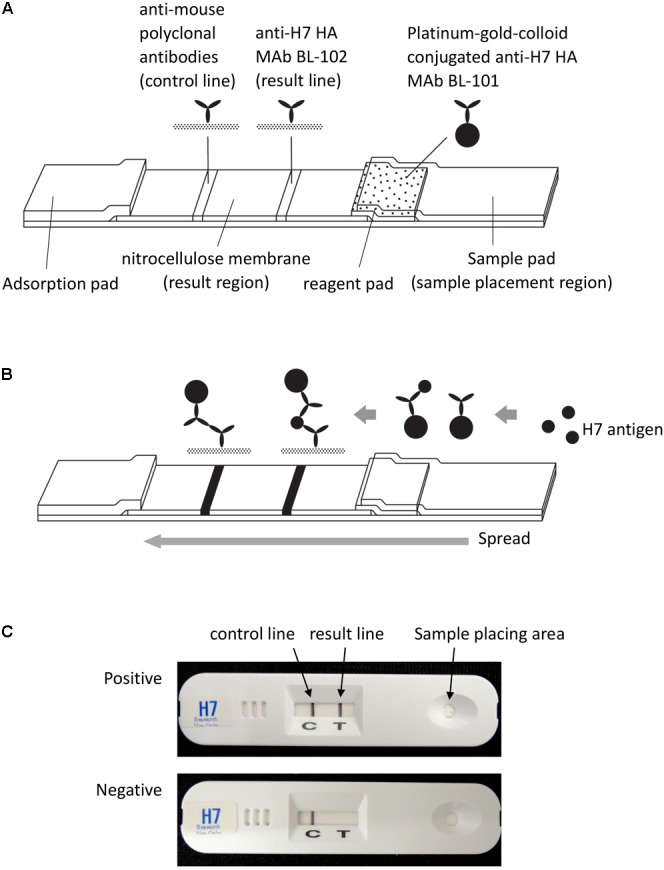
**Schematic representation of the H7 kit. (A)** The kit consists of three regions: a sample placement region, a reagent pad region, and a result region. The reagent pad contains colloidal-platinum-gold-labeled anti-H7 HA MAb BL-101. The result region contains nitrocellulose membrane-immobilized anti-H7 HA MAb BL-102 and anti-mouse immunoglobulin polyclonal antibodies, which serve as capture antibodies in the H7-positive and control lines, respectively. **(B)** When the sample is dropped on the sample pad of the kit, the platinum-gold-colloid-conjugated anti-H7 HA MAb BL-101 dissolves and binds with antigen in the sample. This complex then migrates through the nitrocellulose membrane, and is captured by the immobilized anti-H7 MAb BL-102. Then, the “result” line turns black in the presence of the platinum-gold colloid. Regardless of the presence or absence of H7 HA antigen, the platinum-gold-colloid-conjugated anti-H7 HA MAb BL-101 is captured by the immobilized anti-mouse polyclonal antibodies and produces the control black line. **(C)** Photographs of the H7 kit. Upper image shows an H7-positive result; lower image shows an H7-negative result.

The detection mechanism of the H7 kit is based on an immunochromatographic method that is almost identical to that of the commercially available rapid diagnostic kit ImunoAce Flu (TAUNS Laboratories, Inc., Shizuoka, Japan). ImunoAce Flu uses colloidal-platinum-gold beads, which are platinum-coated colloidal-gold beads; the detection sensitivity of these beads is more than twice that of colloidal-gold beads (Isizuka et al., 2007; Miyashita et al., 2010). Briefly, the kit consists of three regions: a sample placement region, a reagent pad region, and a result region. The reagent pad region contains colloidal-platinum-gold-labeled anti-H7 HA MAbs. The result region contains nitrocellulose membrane-immobilized anti-H7 HA MAbs and anti-mouse immunoglobulin polyclonal antibodies as capture antibodies in the H7-positive and control lines, respectively (**Figure 1**). To develop the kit, two MAbs, BL-101 and BL-102, produced from inactivated Anhui/1-immunized mice were selected on the basis of the result from the immunochromatographic assay. MAb BL-101 was conjugated with platinum-gold-colloid (2.5 μg of the antibody and platinum-gold-colloid complex/kit) to make a complex with the H7 antigens in the samples at the reagent pad region of the kit. MAb BL-102 was immobilized to the nitrocellulose membrane to capture the antibody at the result region (0.2 μg of antibody/kit) (**Figure 1**).

### Specificity and Sensitivity of the H7 Kit Against Various Influenza Virus Subtypes

The specificity and sensitivity of the H7 kit were tested against 15 strains of various subtypes (**Table 1**). All of the viruses reacted with ImunoAce Flu, which can detect type A and B viruses, whereas the H7 kit detected only H7 subtype influenza viruses and did not show any non-specific reactions with any other HA subtypes (**Table 1**). The detection sensitivity of the H7 kit was significantly lower than that of ImunoAce Flu. ImunoAce Flu gave positive results with 10^2^–10^4.7^ TCID_50_ of the viruses, whereas the H7 kit required 10^5.7^–10^7.3^ TCID_50_ of viruses (**Table 1**).

### Detection Sensitivity of the H7 Kit for Recent H7N9 Isolates From Birds and the Environment in China

We analyzed the detection sensitivity of the H7 kit for currently circulating H7N9 viruses in China. Both ImunoAce Flu and the H7 kit gave positive results with all of the viruses tested. The detection limit of ImunoAce Flu ranged from 10^5.0^ to 10^7.0^ EID_50_/100 μl, whereas that for the H7 kit ranged from 10^6.0^ to 10^8.0^ EID_50_/100 μl. The sensitivity of the H7 kit was almost one-tenth lower than that of ImunoAce Flu (**Table 2**).

### Detection Sensitivity of the H7 Kit for Swabs

We also performed a retrospective analysis on frozen swabs from chickens and the surrounding environment that were collected between 2014 and 2015 in China. We selected six frozen swabs from which H7N9 viruses had been previously isolated, and which were positive with ImunoAce Flu. The H7 kit gave positive results with tow out of these swabs (**Table 3**). Both kits gave negative results with six frozen swabs from birds (two from ducks, two from chickens, and two from quails) from which we could not isolate influenza viruses (data not shown). In addition, both kits gave negative results with fresh clinical swabs from two healthy volunteers (data not shown). To examine whether materials in the clinical swabs interfered with the test results, we tested the detection limits of the ImunoAce Flu and H7 kits against A/Guangdong/17SF003/2016 (H7N9) with a fresh swab from a healthy volunteer. We found that the detection limits of both kits were identical to those for the virus alone as described in **Table 1** (data not shown). This result indicates that materials in clinical swabs do not affect the test results.

**Table 3:** Detection sensitivity of the H7 kit for swabs from H7N9-infected birds and the surrounding environment.

ID	Swab from	Ct^a^	Virus^b^	Kit	
				IAce^c^	H7
5	Chicken	22.55	5.59	+	+
15	Environment	26.73	4.31	+	–^d^
16	Environment	26.23	4.46	+	–
17	Chicken	26.14	4.49	+	–
18	Chicken	21.78	5.82	+	+
21	Chicken	25.87	4.57	+	–


*^a^Ct values were measured by use of real-time RT-PCR using H7 primers. ^b^Relative quantification of virus (log_10_ EID_50_/100 μl) in swabs was determined by using the Ct value for each swab and a standard curve (see section “Materials and Methods” for details). ^c^IAce, ImunoAce Flu; ^d^–, not detected.*

To determine the relative quantity of virus in each frozen swab, we performed real-time RT-PCR. Due to the limited amounts of samples available, we were able to perform real-time PCR only once. The cycle threshold (Ct) values of three H7N9 isolates, A/pigeon/Shanghai/S1069/2013, A/chicken/Guangdong/S4021/2014, and A/duck/Shanghai/SD016/2015, were used to generate a standard curve (Supplementary Figure 1), and the relative quantification of virus in each frozen swab was calculated based on the Ct value of each frozen swab by using the standard curve. Frozen swabs containing more than 10^5.59^ EID_50_/100 μl of virus were detected by the H7 kit, and ImunoAce Flu reacted with frozen swabs containing more than 10^4.31^ EID_50_/100 μl (**Table 3**). Although we did not confirm how many live viruses there were in each frozen swab, the detection sensitivity of the H7 kit for viruses in frozen swabs was almost the same as that for isolated viruses (**Tables 2, 3**).

## Discussion

In this study, we developed an influenza diagnosis kit specific for the H7 subtype. Although the detection sensitivity of this H7 kit was almost one-tenth lower than that of ImunoAce Flu, which detects the NP of influenza A or B virus, the kit reacted with only H7 subtypes and did not show any non-specific reactions with other subtypes. The difference in sensitivity between the two kits might be influenced by their different detection antigens: ImunoAce Flu detects NP antigens, whereas the H7 kit detects HA antigens, and the amount of NP in virions and infected cells is higher than that of HA (Robert and Lamb, 2001). The reactivity of the antibodies used in the H7 kit against the virus antigens might also be lower. Modification of the antibodies might therefore improve the sensitivity of the kit.

Manzoor et al. (2008) have also reported the development of a rapid diagnosis kit for H7 avian influenza virus. They used A/duck/Hokkaido/Vac-2/2004 (H7N7) as the antigen in their kit. The detection limits of the Manzoor kit for H7N3 and H7N7 viruses were high (10^3.7^–10^5.3^ EID_50_/test) and almost the same as that of their control kit, Capilia^®^ Flu A+B (10^3.1^–10^4.4^ EID_50_/test) (Manzoor et al., 2008). We previously compared the sensitivity between Capilia Flu A+B and ImunoAce Flu (which we used for comparison with our H7 kit study), and found that the sensitivity of Capilia Flu A+B was 10 times lower than that of ImunoAce Flu (Sakai-Tagawa et al., 2014). This indicates that the sensitivity of Manzoor’s H7 kit is 10 times lower than that of ImunoAce Flu. Since the sensitivity of our H7 kit was also 10 times lower than that of ImunoAce Flu, Manzoor’s kit and our kit may have similar sensitivities against H7 viruses. A direct comparison is needed to further evaluate these differences in detection sensitivity. Jin et al. (2014) and Kang et al. (2014) also developed immunochromatographic tests for rapid diagnosis using anti-H7 MAbs immediately after the first wave of LPAI H7N9; the sensitivity of those tests against LPAI H7N9 viruses was higher than ours. However, since 2013, the H7N9 viruses have antigenically evolved with substantial mutations in their HA protein (World Health Organization, 2017a; Zhu et al., 2017). Here we show that our kit detects not only LPAI but also HPAI H7N9 strains; whether the kits developed by Manzoor et al. (2008), Jin et al. (2014), and Kang et al. (2014) detect HPAI H7N9 strains remain unknown. We therefore believe our kit will be useful for monitoring H7N9 virus outbreaks.

In our retrospective analysis of six H7N9-positive frozen swabs, the H7 kit detected virus in two of these six swab samples that were positive with ImunoAce Flu (**Table 3**). Although the sensitivity of the H7 kit was 10 times lower (>10^5.59^ EID_50_/100 μl) than that of ImunoAce Flu (>10^4.31^ EID_50_/100 μl) (**Table 3**), these results demonstrate that the H7 kit could be used to detect H7 virus in swabs from patients. In addition, this H7 kit can detect viruses of different H7 subtypes (**Table 1**). This H7 kit will therefore be useful not only for analyzing human samples in the clinical setting but also in the field of veterinary medicine for earlier detection of HPAI H7 viruses. We tested only one H7N2 and one H7N7 virus and did not test other H7 viruses. Therefore, although this H7 kit has potential as a diagnostic kit for H7 viruses in general, its ability to detect H7 viruses other than H7N9 viruses needs to be tested in the future. In addition, identification of the epitopes of the MAbs used in our kit will help us to understand the potential changes in the sensitivity of the kit to H7 HA as the virus continues to change.

Since 2016, human infections with HPAI H7N9 viruses have been detected in China (World Health Organization, 2017b). To date, the H7N9 viruses have not caused a pandemic because of their limited human-to-human transmissibility. However, we found that HPAI H7N9 viruses can transmit among ferrets (Imai et al., 2017). Moreover, there have now been many reports of mammalian-adaptive mutations in H7N9 viruses that could confer pandemic potential (Wang D. et al., 2014; Wang Y.R. et al., 2014; Watanabe et al., 2014; Xiao et al., 2016). Prompt identification and isolation of H7N9 patients would help prevent the spread of H7N9 viruses and potentially a pandemic. Although improvements to optimize its sensitivity are required, the H7 kit developed in this study represents a valuable tool for the early detection of H7N9-infected patients.

## Author Contributions

KI-H, JS, XW, YS-T, MI, KM, KN, SY, SW, HC, and YK conceived the experiments. KI-H, JS, XW, YS-T, MI, KM, TSL, KN, SY, and SW conducted the experiments. KI-H and YK wrote the manuscript. All authors reviewed the manuscript.

## Conflict of Interest Statement

YK has received speaker’s honoraria from Toyama Chemical Co., Ltd. and Astellas Pharma Inc., has received grant support from Chugai Pharmaceutical Co., Ltd., Daiichi Sankyo Company, Limited, Toyama Chemical Co., Ltd., TAUNS Laboratories, Inc., Otsuka Pharmaceutical Co., Ltd., and Denka Seiken Co., Ltd., and is a co-founder of FluGen Inc. KM, KN, and SW are employed by TAUNS Laboratories, Inc. TAUNS Laboratories, Inc. has patents “Japan patent JP3886000B” and “United States patent US7713328.” The remaining authors declare that the research was conducted in the absence of any commercial or financial relationships that could be construed as a potential conflict of interest.
