# Supplementary material for: Development of an Influenza Rapid Diagnostic Kit Specific for the H7 Subtype
Source: Front Microbiol. 2018 Jun 25;9:1346. doi: 10.3389/fmicb.2018.01346 (PMC6026626; doi:10.3389/fmicb.2018.01346)
Supplement: Supplementary file 1 [file Image_1.PDF]

Supplementary Figure 1. Standard curve for determining the relative quantity of virus in each swab. (a) Raw Ct values (triplet) of three H7N9 isolates, A/pigeon/Shanghai/S1069/2013, A/chicken/Guangdong/S4021/2014, and A/duck/Shanghai/SD016/2015. (**b)** Standard curve produced from the average Ct values of the three isolates.

(a)

EID_{50}/ 100 µl	Ct value									
	PG/SH/S1069/2013(H7N9)			CK/GD/S4021/2014(H7N9)			DK/SH/SD016/2015(H7N9)			Average
	1	2	3	1	2	3	1	2	3	
10^{0}	35.000	35.000	35.000	35.000	35.000	35.000	35.000	35.000	35.000	35.000
10^{1}	35.000	35.000	35.000	35.000	35.000	35.000	35.000	35.000	35.000	35.000
10^{2}	35.000	35.000	35.000	35.000	35.000	35.000	35.000	35.000	35.000	35.000
10^{3}	31.510	31.070	31.150	30.700	30.540	30.580	31.280	31.130	31.030	30.999
10^{4}	27.820	27.800	27.850	27.230	27.320	27.380	28.000	28.010	27.980	27.710
10^{5}	24.450	24.620	24.640	24.010	23.870	24.000	24.930	25.000	24.780	24.478
Negative control	35.000	35.000	35.000	35.000	35.000	35.000	35.000	35.000	35.000	35.000

(b)

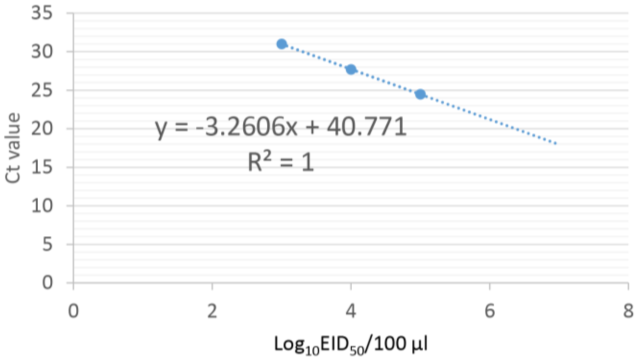

Supplementary figure 1, Iwatsuki-Horimoto et al.
