# Supplementary material for: Development of an Influenza Rapid Diagnostic Kit Specific for the H7 Subtype
Source: Front Microbiol. 2018 Jun 25;9:1346. doi: 10.3389/fmicb.2018.01346 (PMC6026626; doi:10.3389/fmicb.2018.01346)
Supplement: Supplementary file 2 [file Table_1.DOCX]

Supplementary Table 1. Purified HA proteins used to screen hybridomas by ELISA.

| Subtype | Strain | Company | ID | Product name |
| --- | --- | --- | --- | --- |
| B/Victoria lineage | B/Ohio/1/2005 | BEI Resources | NR-19243 | Hemagglutinin (HA) Protein from Influenza Virus, B/Ohio/1/2005, Recombinant from Baculovirus -- Influenza B virus (Victoria lineage) |
| H1N1  (pdm09) | A/California/07/2009 | Sino Biological | 11085-V08H | Influenza A H1N1 (A/California/07/2009) Hemagglutinin |
| H2N2 | A/Canada/720/2005 | Sino Biological | 11688-V08H | Influenza A H2N2 (A/Canada/720/2005) Hemagglutinin |
| H3N2 | A/Perth/16/2009 | Sino Biological | 40043-V08H | Influenza A H3N2 (A/Perth/16/2009) Hemagglutinin Protein (His Tag) |
| H5N1 | A/Indonesia/5/2005 | Sino Biological | [11060-V08H1](http://jp.sinobiological.com/H5N1-Hemagglutinin-g-616.html) | Influenza A H5N1 (A/Indonesia/5/2005) Hemagglutinin / HA Protein (His Tag) |
| H6N1 | A/northern shoveler/California/HKWF115/2007 | Sino Biological | 11723-V08H | Influenza A H6N1 (A/northern shoveler/California/HKWF115/2007) Hemagglutinin |
| H7N9 | A/Anhui/1/2013 | Sino Biological | 40103-V08H | Influenza A H7N9 (A/Anhui/1/2013) Hemagglutinin / HA Protein (His Tag) |
| H9N2 | A/Hong Kong/35820/2009 | Sino Biological | 40174-V08B | Influenza A H9N2 (A/Hong Kong/35820/2009) HA / Hemagglutinin Protein (His Tag) |
